# Radiological features of ankle synovial cell sarcoma

**DOI:** 10.11604/pamj.2021.38.301.27274

**Published:** 2021-03-23

**Authors:** Jihane Habi, Nadia Moussali

**Affiliations:** 1 Department of Radiology, Faculty of Medicine, Mohammed VI University of Health Sciences/Cheikh Khalifa International University Hospital, Casablanca, Morocco,; 2 20 Aout Radiology Department of the Ibn Rochd of Casablanca University, Casablanca, Morocco

**Keywords:** Imaging, synovial sarcoma, ankle

## Image in medicine

A 23-years-old man was referred to our institution to investigate a painless right ankle mass. The clinical examination found a painful mass, fixed on the superficial and deep plane with vascular turgor. It measured 15x10cm (A). The X-ray of the ankle was done first and revealed opacity centred on the soft external malleolar tissues (arrow B). Then a magnetic resonance imaging (MRI) was performed. It showed multi lobulated mass with heterogeneous enhancement after injection of gadolinium (C) and a specific triple signal in T2 weighted images (D), made by a hyper signal (liquid), intermediate signal (tumor tissue) and hypo signal (septa). This radiological finding reminds a “bowl of grapes”. The extension to the carpal bones was diagnosed as a hyper signal area on T2 (arrow D). The diagnosis of synovial sarcoma was suggested and then confirmed by pathological analysis after performing a surgical biopsy. The patient has developed a metastatic location in the right thigh. Synovial sarcoma is a malignant mesenchymal tumor. It presents 10% of all ankle soft tumors, and affects young adults with a sex ratio of 1. The lesion is often slow growing. On MRI, the synovial sarcoma diagnosis strongly suggestive by the classic “triple sign”; and “bowl of grapes sign” in T2 sequences. Its treatment is based on surgical excision adjunctive with chemotherapy or radiotherapy.

**Figure 1:**
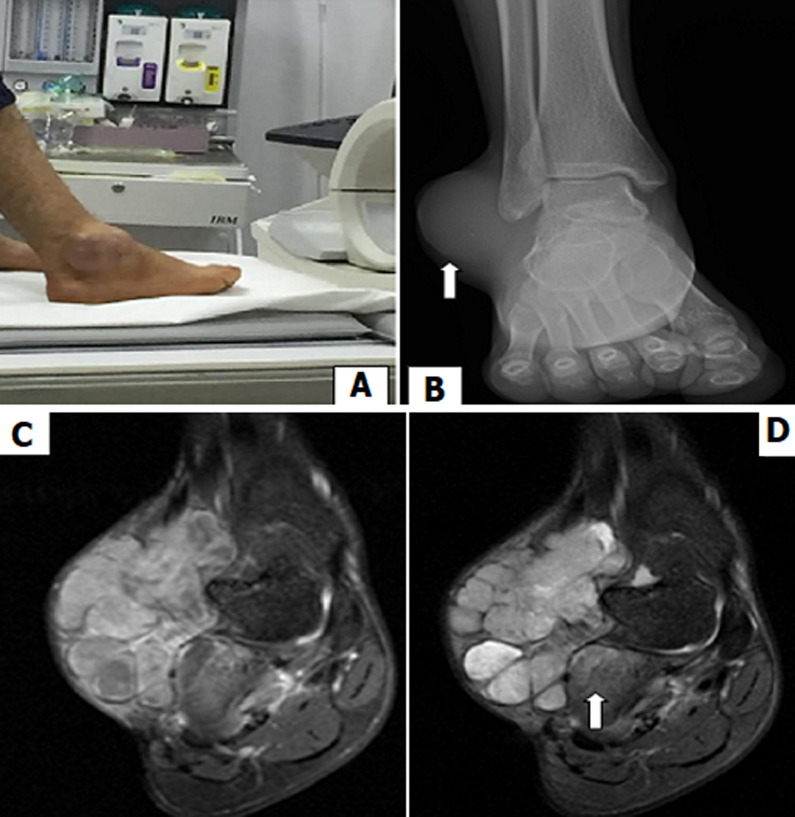
illustration of ankle synovial sarcoma (A); radiographic X-ray (B); magnetic resonance imaging (C, D)

